# Global research trends and hotspots in cancer-related anemia from 2010 to 2025: a multidatabase bibliometric analysis

**DOI:** 10.3389/fonc.2026.1971034

**Published:** 2026-10-28

**Authors:** Longjun Huang, Luyang Chi, Jiahao Sheng, Li Hou, Huafang Lin, Huaifang Fan

**Affiliations:** 1 Beijing University of Chinese Medicine, Beijing, China; 2 Dongzhimen Hospital, Beijing University of Chinese Medicine, Beijing, China; 3 First School of Clinical Medicine, Zhejiang Chinese Medical University, Hangzhou, Zhejiang, China; 4 Hangzhou Red Cross Hospital, Hangzhou, Zhejiang, China

**Keywords:** bibliometric analysis, cancer-related anemia, erythropoiesis-stimulating agents, iron deficiency, knowledge mapping, research hotspots

## Abstract

**Background:**

Cancer-related anemia (CRA) is the most common hematologic complication of malignant tumors and their treatments, affecting treatment delivery, quality of life and survival. A macro-level map of the field’s knowledge structure, evolution and frontiers is still lacking.

**Methods:**

Publications on CRA published between January 1, 2010 and December 31, 2025 were retrieved from the Web of Science Core Collection (WoSCC) and PubMed using an explicit, reproducible query adapted to each database. Of 2,568 records (WoSCC, n = 1,110; PubMed, n = 1,458), 746 articles and reviews met the inclusion criteria after database-specific screening, deduplication and record-level verification;509 records were indexed in both databases, 55 were retrieved from WoSCC only and 182 from PubMed only. Citation counts came from OpenAlex. The analysis covers the English-language literature indexed in these two databases. Publication trends, leading countries/regions, institutions, authors and journals, citation indicators and keyword networks were analyzed to characterize temporal dynamics and emerging research fronts.

**Results:**

Annual output was stable at about 45 publications per year between 2010 and 2024 (no trend; annual rate ratio 1.00, 95% CI 0.98–1.02) and reached 70 publications in 2025, a single-year increase that exceeds the value predicted from the 2010–2024 trend. The United States (n = 208, 27.9%), China (n = 105) and the United Kingdom (n = 77) were the most productive countries, while year-normalized citation impact was highest for Switzerland (1.72, 95% CI 1.15–2.37), Austria (1.69) and Italy (1.58). Amgen was the leading institution and Aapro M the most productive author. Supportive Care in Cancer (n = 44) was the most prolific journal and Annals of Oncology had the highest normalized citation score (3.99, 95% CI 2.07–6.00).

**Conclusion:**

CRA research output was stable between 2010 and 2024 apart from a single-year increase in 2025. As reflected in keyword frequencies rather than in clinical practice, research attention moved from erythropoiesis-stimulating agent-centered topics toward iron metabolism, perioperative anemia and outcome-oriented research. Strengthening international collaboration and translating iron-regulatory and inflammatory mechanisms into clinical strategies are key directions for future research.

## 1 Introduction

Cancer-related anemia (CRA) is the most common hematologic complication in patients with malignant tumors, a reduction in red blood cell mass or hemoglobin level caused by the malignancy itself or by anticancer treatment (1). Its pathogenesis is multifactorial. Beyond direct bone marrow infiltration, tumor-associated blood loss and hemolysis, the tumor microenvironment releases inflammatory cytokines that disrupt iron metabolism, suppress endogenous erythropoietin (EPO) production and blunt the response of bone marrow erythroid progenitors to EPO; together, these effects drive ineffective erythropoiesis (2, 3). Anticancer therapies can also aggravate anemia through myelosuppression, nephrotoxicity, and nutritional impairment (4, 5).

CRA is highly prevalent and clinically consequential. A systematic scoping review covering 65,179 patients across five continents reported CRA prevalence ranging from 12.8% to 100%, depending on tumor type, stage, treatment and patient characteristics (6). The European Cancer Anemia Survey (ECAS) showed that approximately 39% of patients with solid tumors already had baseline anemia before treatment, that anemia developed in up to 63% of patients after chemotherapy, and that severity increased with successive treatment cycles (7). In China, a multicenter survey of 7,324 patients with malignant tumors reported an overall CRA prevalence of 49.24%, with mild anemia accounting for 28.84% and moderate-to-very-severe anemia for 20.41% (8). Despite guideline recommendations, more than half of anemic cancer patients do not receive standardized anemia assessment and treatment, and mild-to-moderate anemia is particularly prone to underdiagnosis and undertreatment (9).

The clinical importance of CRA extends beyond hematologic indices. Anemia-related fatigue, dyspnea, and cognitive dysfunction impair daily function and quality of life, and fatigue has been recognized as one of the most distressing symptoms in oncology (10). CRA also compromises the delivery of anticancer therapy: moderate-to-severe anemia frequently necessitates chemotherapy dose reduction or treatment delay, and anemia-induced tumor hypoxia can promote aggressive phenotypes and reduce the efficacy of radiotherapy and immune checkpoint inhibitors (11, 12). Effective management of CRA, through erythropoiesis-stimulating agents (ESAs), intravenous iron, transfusion support and, more recently, hypoxia-inducible factor prolyl hydroxylase inhibitors (HIF-PHIs), is central to supportive oncology care (5, 13, 14).

Over the past decade, research on CRA has expanded substantially, spanning iron-regulatory mechanisms, novel iron formulations, ESA safety, patient blood management (PBM), and anemia in the era of immunotherapy (15–17). However, existing reviews are largely mechanism- or treatment-specific, and no study has yet mapped the CRA literature indexed in these two databases at a macro level, covering its knowledge structure, evolution, hotspots and collaboration patterns. Bibliometric analysis uses mathematical and statistical methods to quantitatively map large volumes of publication metadata, thereby complementing narrative reviews by revealing field-level trends, core contributors, intellectual bases and research frontiers (18). In this study, we performed a comprehensive bibliometric analysis of CRA publications indexed in the Web of Science Core Collection and PubMed between 2010 and 2025, integrated with citation data from OpenAlex, to (i) characterize publication output and growth in this corpus; (ii) identify leading countries, institutions, authors and journals; (iii) map collaboration networks and keyword co-occurrence structures; and (iv) track temporal dynamics and emerging research frontiers. This analysis complements previous reviews by mapping how the field is organized and how it has changed. Its contribution lies in a corpus harmonized from two databases with per-stage reporting, in citation indicators adjusted for publication year, and in research fronts that are tied to the underlying keyword evidence rather than to clinical practice.

## 2 Materials and methods

### 2.1 Data sources and search strategy

We searched the Web of Science Core Collection (WoSCC) and PubMed, covering the period from January 1, 2010 to December 31, 2025, restricted to English-language articles and reviews; two records indexed as conference proceedings papers in the source databases but otherwise meeting the inclusion criteria were retained after manual review and classified as articles. The following explicit topic query was used (search fields: [tiab] = title/abstract; [ti] = title):

((“cancer-related anemia”[tiab] OR “cancer associated anemia”[tiab] OR “cancer-associated anemia”[tiab] OR “cancer-related anaemia”[tiab] OR “cancer associated anaemia”[tiab] OR “tumor-related anemia”[tiab] OR “tumour-related anemia”[tiab] OR “tumour-related anaemia”[tiab] OR “malignancy-related anemia”[tiab] OR “malignancy-associated anemia”[tiab] OR “anemia of malignancy”[tiab] OR “anaemia of malignancy”[tiab] OR “anaemia of cancer”[tiab] OR “chemotherapy-induced anemia”[tiab] OR “chemotherapy induced anemia”[tiab] OR “chemotherapy-induced anaemia”[tiab] OR “chemotherapy induced anaemia”[tiab] OR “anemia in cancer patients”[tiab] OR “anaemia in cancer patients”[tiab] OR “cancer anemia”[tiab] OR “cancer anaemia”[tiab] OR “tumor anemia”[tiab] OR “tumour anaemia”[tiab]) OR ((anemi*[ti] OR anaemi*[ti]) AND (cancer*[ti] OR tumor*[ti] OR tumour*[ti] OR neoplasm*[ti] OR malignan*[ti] OR chemotherap*[ti] OR oncolog*[ti] OR carcinom*[ti] OR “hematologic malignancy”[ti] OR “haematologic malignancy”[ti]))) AND (“2010/01/01”[dp]: “2025/12/31”[dp]) AND english[la] AND (“Journal Article”[pt] OR “Review”[pt]).

The equivalent Web of Science Core Collection query (TS, topic; TI, title) was: TS=(“cancer-related anemia” OR “cancer associated anemia” OR “cancer-associated anemia” OR “cancer-related anaemia” OR “cancer associated anaemia” OR “tumor-related anemia” OR “tumour-related anemia” OR “tumour-related anaemia” OR “malignancy-related anemia” OR “malignancy-associated anemia” OR “anemia of malignancy” OR “anaemia of malignancy” OR “anaemia of cancer” OR “chemotherapy-induced anemia” OR “chemotherapy induced anemia” OR “chemotherapy-induced anaemia” OR “chemotherapy induced anaemia” OR “anemia in cancer patients” OR “anaemia in cancer patients” OR “cancer anemia” OR “cancer anaemia” OR “tumor anemia” OR “tumour anaemia”) OR (TI=(anemi* OR anaemi*) AND TI=(cancer* OR tumor* OR tumour* OR neoplasm* OR malignan* OR chemotherap* OR oncolog* OR carcinom* OR “hematologic malignancy” OR “haematologic malignancy”)), restricted to English-language articles and reviews published between 2010 and 2025.

The query deliberately avoided bare acronyms (e.g., “CRA”, “CIA”) to prevent false-positive retrieval of unrelated entities (e.g., colorectal adenoma, collagen-induced arthritis, cell-mediated immunity, chemical index of alteration).

The search retrieved 2,568 records (WoSCC, n = 1,110; PubMed, n = 1,458). Records were screened in three predefined stages, each documented in a screening log that reports the rules applied, the number of records removed at each step with the reason, and the near-duplicate pairs examined (**Supplementary File S1**). In Stage 1 the two databases were screened separately by two reviewers working independently and applying identical criteria. Step 1a re-checked the document-type, language and publication-year limits (10 WoSCC and 19 PubMed records removed); step 1b assessed the remaining 1,100 WoSCC and 1,439 PubMed records on title and abstract against the criteria in Section 2.2. Exclusions in step 1b were 535 in WoSCC (287 distinct hematologic entities; 248 incidental, off-topic or non-article records) and 545 in PubMed (404 and 141, respectively), leaving 1,459 records (WoSCC, n = 565; PubMed, n = 894; **Supplementary Table S1**). Disagreements were resolved by discussion or, where agreement could not be reached, by a third senior author, and records that could not be classified with certainty from the title and abstract were flagged for full-record assessment in Stage 3 rather than excluded.

In Stage 2, duplicates were removed with a four-step algorithm implemented in Python: exact matching on the normalized DOI, the PubMed identifier and the WoSCC accession number, followed, for records without these identifiers or still unmatched after identifier matching, by a normalized title + first author + year key with token-based title comparison (>= 0.95 duplicates; 0.85-0.95 manual review; < 0.85 distinct, listed in the log with the reason). This removed 509 duplicates (505 by PubMed identifier and 4 by DOI); the title step returned no further duplicates but was applied to the whole set because 45 of the 746 included records (6.0%) have no DOI and 12 (1.6%) have no PubMed identifier. In Stage 3, every remaining record was checked against the complete record, because document-type labels are unreliable in both databases: PubMed indexes many letters and case reports as “Journal Article”, and WoSCC labels some correspondence and case reports as “Article”. This removed 204 records (47 case reports identified only from the PubMed document-type field, 2 letters or other non-article items, and 155 records already excluded in the corresponding copy of the other database). The final corpus comprised 746 records published between 2010 and 2025 (632 articles, including two indexed as conference proceedings papers, and 114 reviews): 509 records were indexed in both databases, 55 were retrieved from WoSCC only and 182 from PubMed only. **Figure 1** shows the flow of records through the three stages, separately for the two databases.

**Figure 1:**
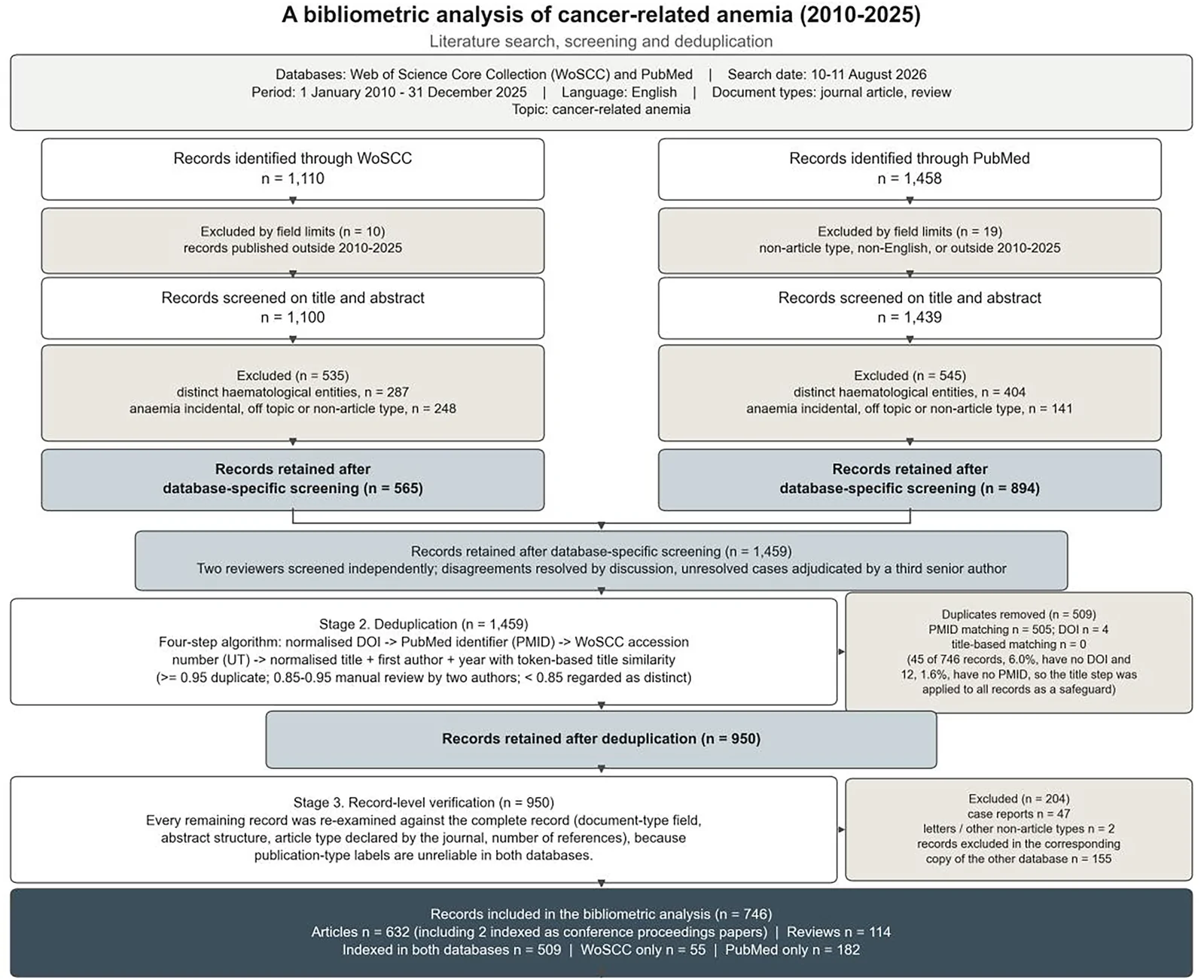
Flowchart of the literature search, screening and deduplication. Records identified in the Web of Science Core Collection (WoSCC) and PubMed were screened separately by two independent reviewers against the document-type, language and publication-year limits (step 1a) and then on title and abstract against the inclusion and exclusion criteria (step 1b). The records retained by the two databases were merged, deduplicated with a four-step algorithm (identifier matching followed by title-based matching) and verified at the record level before the final corpus was assembled. The number of records entering and leaving each stage, separately for each database and with the reason for exclusion, is given in **Supplementary Table S1**; the screening workflow, exclusion reason codes and the near-duplicate pairs examined are documented in **Supplementary File S1**. WoSCC, Web of Science Core Collection; CRA, cancer-related anemia; PMID, PubMed identifier; UT, Web of Science accession number.

### 2.2 Inclusion and exclusion criteria

Records were included if they were journal articles or reviews published in English between 2010 and 2025 whose topic was CRA (anemia caused by malignancy or anticancer therapy, or the clinical management of such anemia). Records focusing on distinct hematologic entities that are not CRA, including Fanconi anemia, aplastic anemia, pernicious anemia, sickle cell disease, thalassemia, autoimmune hemolytic anemia, paroxysmal nocturnal hemoglobinuria and chronic kidney disease-related anemia, were excluded. Records in which anemia was incidental (e.g., anemia as a side effect in general oncology studies, veterinary studies, erythrocyte biology without cancer context, or unrelated screening and diagnostic algorithms) were also removed after full title and abstract screening. Case reports, letters, editorials, conference abstracts and retracted articles were excluded, consistent with the search restriction to journal articles and reviews; as noted in Section 2.1, two records indexed as conference proceedings papers were retained as articles after manual review. Both are full-length articles published in indexed journals that also carry the WoS document-type tag “Proceedings Paper” because their content was presented at a meeting; neither is a conference abstract. Their document-type fields are given in **Supplementary Data S2** (sheet 1). Document-type limits were applied both in the database query and at the record level in Stage 3, because publication-type labels are not assigned consistently by either database and residual non-article document types can therefore survive an initial filter; the number of records removed at each of these two points is reported separately in Section 2.1 and in **Supplementary Table S1**. The screening process is summarized in **Figure 1**.

### 2.3 Data extraction and normalization

Bibliographic records (title, abstract, author keywords, Medical Subject Headings [MeSH], authors, affiliations, journal, year, DOI) were parsed from the Web of Science plain-text records and PubMed XML; Keywords Plus fields, which are generated only for Web of Science records, were parsed when available. Citation counts were retrieved from OpenAlex by DOI (with PMID fallback) on 24 September 2026 (19). 745 of the 746 records were matched, and the one record that carries neither a DOI nor a PubMed identifier is excluded from the citation-based analyses. Countries and institutions were assigned primarily from OpenAlex authorship metadata (country codes and institution display names) and supplemented by parsing PubMed affiliation strings when OpenAlex data were unavailable. Author names were standardized to OpenAlex author entities, and name variants of the same person (for example, different given-name initials or name suffixes) were merged under a manually verified dictionary (**Supplementary Data S2**, **sheet 4**). Duplicate records were removed with the four-step algorithm described in Section 2.1. Of the 509 duplicates removed, 505 were detected by PubMed identifier matching and 4 by DOI matching; no additional duplicates were detected by title-based matching, which was applied to the whole set because 45 of the 746 included records (6.0%) have no DOI, 12 (1.6%) have no PubMed identifier and 2 (0.3%) carry neither identifier. Because identifier-based deduplication cannot detect dual publication of identical content under different DOIs (e.g., the ASCO/ASH 2010 guideline published in both the Journal of Clinical Oncology and Blood), such records may be retained as separate entries; this is disclosed in the corresponding results text and figure legend.

### 2.4 Bibliometric and network analyses

Descriptive bibliometrics included annual publication counts, publication and citation counts by country, institution, author and journal, and author h-index. Country-level publication and citation counts used whole counting, in which a publication co-authored by authors from multiple countries was credited to each contributing country. Collaboration networks were constructed for countries/regions, institutions and authors, with edge weights equal to the number of shared documents; clusters in the VOSviewer maps are those detected by the VOSviewer clustering algorithm. For the VOSviewer keyword analyses, author keywords and MeSH descriptors were combined after excluding generic indexing terms (e.g., “humans”, “female”, “male”, “adult”, “retrospective studies”) and after applying the keyword harmonization thesaurus (**Supplementary Data S2**, **sheet 5**); co-occurrence strength was measured by association strength. Keywords occurring at least five times were retained, and the largest connected component of the resulting network was clustered. Temporal dynamics were characterized by period-normalized keyword frequencies across 2010–2013, 2014–2017, 2018–2021 and 2022–2025, and emerging fronts were defined as keywords whose share of occurrences in 2022–2025 was at least 1.6 times their share in 2010–2021, with at least four occurrences after 2021 so that single-record terms could not qualify. The 1.6-fold cut-off was set before the analysis as a clear relative gain in research attention; because any cut-off is a judgement, the selection was repeated at 1.5, 2.0, 2.5 and 3.0 and the results are reported in **Supplementary Table S2**, and the resulting fronts should be interpreted as indicative rather than definitive. All quantitative analyses were performed in Python (version 3.12) using NetworkX and Matplotlib, and network visualizations were generated with VOSviewer (1.6.20) and CiteSpace (6.4.R1). To align the visualizations with the standards of the science-mapping literature, network and temporal maps were also generated with VOSviewer (20) and CiteSpace (21) from the 746-record corpus. VOSviewer was used for co-authorship networks of countries/regions (≥ 5 documents), institutions (≥ 5 documents) and authors (≥ 3 documents), and for keyword co-occurrence networks (≥ 5 occurrences; association-strength normalization), with network, density and overlay (average publication year) views. CiteSpace was used with one-year time slices over 2010–2025. The CiteSpace keyword timeline and burst analyses used the harmonized 238-keyword list. One-year time slices were used with the g-index criterion (k = 15), LRF = 2.5, L/N = 10, LBY = 5 and e = 1.0, with Pathfinder pruning. The resulting keyword network contained 238 nodes and 383 links, with modularity Q = 0.733 and weighted mean silhouette S = 0.903.The reference co-citation network used the g-index criterion with k = 5, LRF = 2.5, L/N = 10, LBY = 5 and e = 1.0 with Pathfinder pruning (184 nodes, 244 links; modularity Q = 0.741, weighted mean silhouette S = 0.906). Bursts were detected for references and keywords with the default settings (γ = 1.0; minimum duration two years). Annual publication counts were analyzed with a Poisson regression of the annual count on the publication year and compared with a negative binomial model because the counts were mildly overdispersed, and period means were compared with a Poisson model using period indicators; cumulative publication curves are presented for description only. Citation indicators are reported both as raw means and as age-adjusted values (citations per year since publication; year-normalized citation score, NCS = observed citations divided by the mean citations of all corpus records published in the same year) together with medians and 95% bootstrap confidence intervals, and entities with fewer than five publications are not ranked.

### 2.5 Ethics

This study used only publicly available bibliographic metadata and did not involve human subjects; therefore, ethics approval was not required.

## 3 Results

### 3.1 Publication trends

A total of 746 unique publications met the inclusion criteria, comprising 632 articles (84.7%, of which two were indexed as conference proceedings papers) and 114 reviews (15.3%). These records accumulated 17,875 citations (mean 24.0, median 11); 55 of the 745 records matched in OpenAlex had not been cited. As shown in **Figure 2**, annual output showed no detectable trend: averaged over 2010–2024 the corpus contributed 45.1 publications per year (SD 6.3, range 34–58), and a Poisson regression of the annual count on the publication year gave an annual rate ratio of 1.00 (95% CI 0.98–1.02; p = 0.97), with an equivalent negative binomial fit (p = 0.98). Output in 2016–2024 averaged 42.9 publications per year, 11% lower than the 2010–2015 mean of 48.3 (rate ratio 0.89, 95% CI 0.76–1.03; p = 0.12). The only significant feature of the series is 2025, with 70 publications, which is 1.55 times the 2010–2024 mean and exceeds the value predicted from the 2010–2024 trend (39.8, 95% CI 33.8–46.9; p < 0.001). Because a cumulative curve increases monotonically by construction, the cumulative series in **Figure 2** is descriptive and is not used as evidence of growth. The 2025 increase may reflect either accelerated research activity or year-specific indexing and publishing dynamics and should be confirmed as additional late-2025 and 2026 records are indexed.

**Figure 2:**
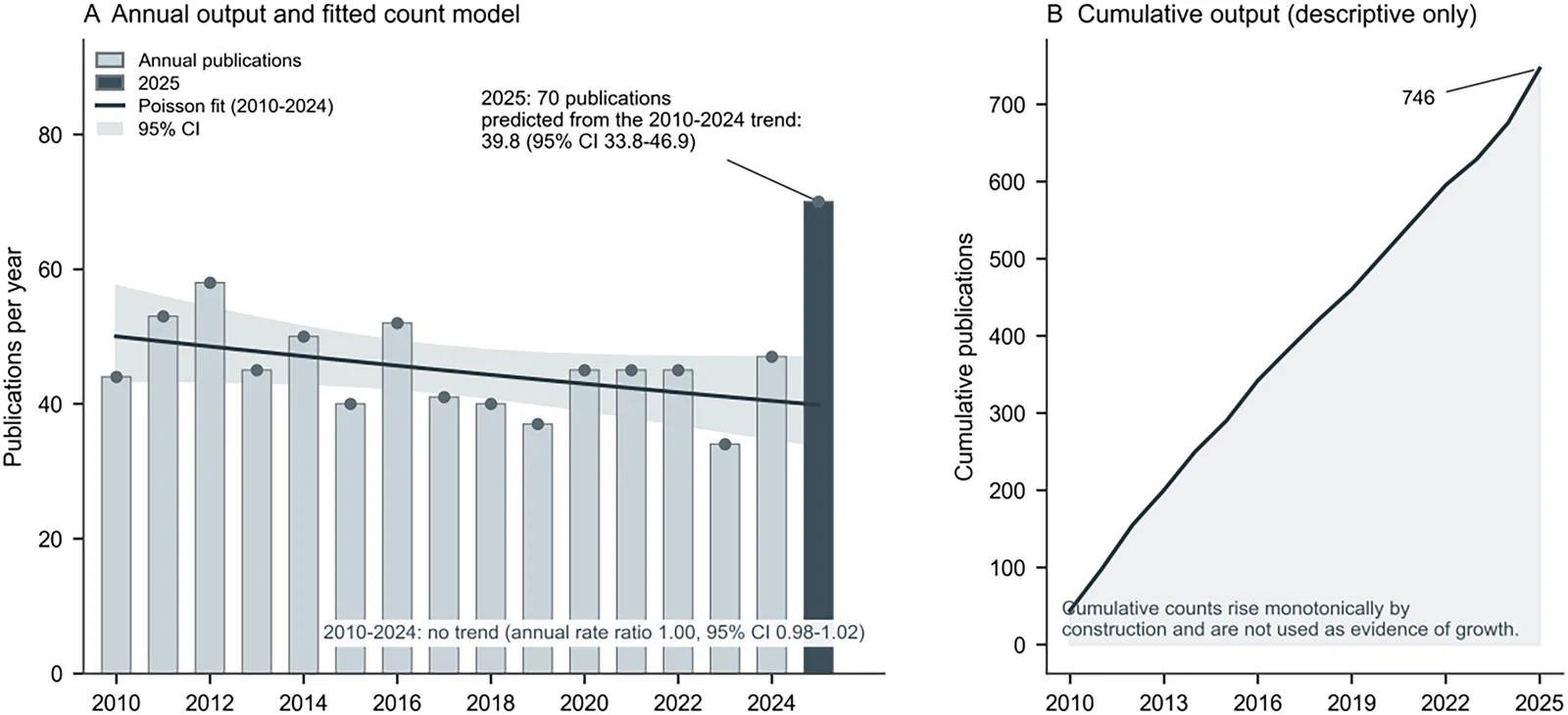
Annual output and cumulative output of cancer-related anemia publications, 2010-2025 (n = 746). **(A)** Annual number of publications, with the fitted mean for 2010-2024 (45.1 publications/year) and its 95% confidence interval from the Poisson count model; the 2025 value is a single-year observation and should not be interpreted as evidence of a sustained trend. **(B)** Cumulative number of publications; cumulative counts increase monotonically by construction and are presented for descriptive purposes only, not as evidence of growth.

### 3.2 Countries/regions

Publications were contributed by authors from 73 countries/regions. **Figure 3A** is a bar chart showing the number of publications by country, with the United States, China and the United Kingdom ranking as the top three in terms of publication volume. To improve readability while preserving the principal collaborative structure, the country/region co-authorship network in **Figure 3B** was restricted to countries/regions with at least five publications (N = 28). The United States (n = 208) was the largest node and principal collaboration hub, followed by China (n = 105) and the United Kingdom (n = 77). Germany (n = 64), Italy (n = 57), Switzerland (n = 50) and Spain (n = 46) formed additional connected hubs, whereas countries with smaller publication volumes occupied more peripheral positions in the network. (**Table 1**). The network displayed a multicenter, cluster-based structure. The densest collaborations involved the United States, the United Kingdom and several European countries, whereas China and other Asia-Pacific countries occupied a separate but connected segment of the network. Node color denotes membership of a VOSviewer collaboration cluster and does not encode publication year. The five-publication threshold was applied only for visualization and did not alter the underlying country/region calculations.

**Figure 3:**
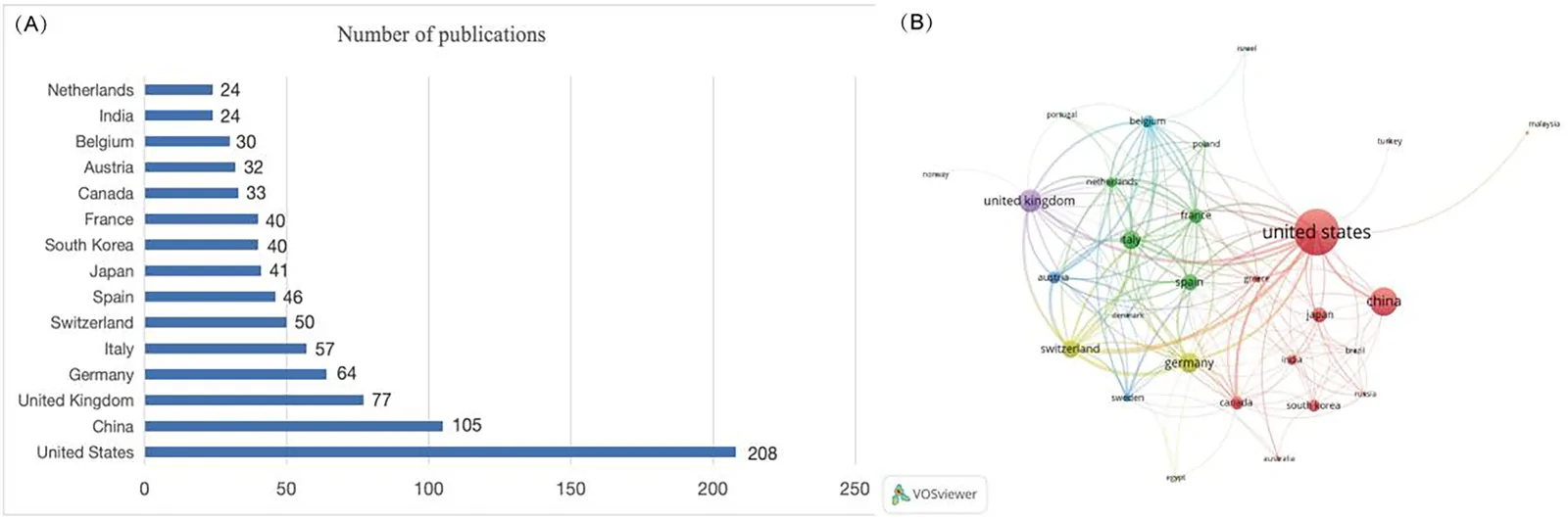
Geographic distribution of cancer-related anemia research. **(A)** Top 15 countries/regions by publication output. **(B)** Country/region co-authorship network constructed with VOSviewer, including countries/regions with at least five publications (N = 28). Node size is proportional to publication output, node color denotes a VOSviewer collaboration cluster, and edge thickness represents co-authorship link strength. The publication threshold was applied only to improve legibility.

**Table 1:** Top 15 countries/regions by publication output on cancer-related anemia (2010-2025), with age-adjusted citation indicators.

Rank	Country/region	Publications	Share (%)	Citations (OpenAlex)	Mean citations	Median citations	Citations per year	NCS	NCS CI low	NCS CI high	SCP	MCP
1	United States	208	27.9	7112	34.2	14	3.38	1.32	1.06	1.61	105	103
2	China	105	14.1	2031	19.3	11	2.6	0.96	0.74	1.21	85	20
3	United Kingdom	77	10.3	2506	32.5	15	3.22	1.08	0.76	1.5	35	42
4	Germany	64	8.6	2061	32.2	14	2.9	1.1	0.72	1.56	21	43
5	Italy	57	7.6	2403	42.2	16	4.65	1.58	1.1	2.16	31	26
6	Switzerland	50	6.7	2755	55.1	17	4.83	1.72	1.15	2.37	5	45
7	Spain	46	6.2	1377	29.9	14	2.96	1.04	0.63	1.54	23	23
8	Japan	41	5.5	641	15.6	8	1.93	0.84	0.51	1.28	31	10
9	France	40	5.4	543	13.6	11.5	1.34	0.54	0.38	0.74	21	19
10	South Korea	40	5.4	804	20.1	14	2.24	0.87	0.64	1.11	34	6
11	Canada	33	4.4	1274	38.6	12	3.92	1.52	0.99	2.1	16	17
12	Austria	32	4.3	1688	52.8	11	4.64	1.69	0.9	2.61	6	26
13	Belgium	30	4	1211	40.4	12	3.16	1.18	0.64	1.88	9	21
14	India	24	3.2	453	18.9	5.5	2.33	0.91	0.41	1.52	18	6
15	Netherlands	24	3.2	677	28.2	22	3.22	1.08	0.62	1.71	5	19

Countries are assigned from OpenAlex authorship metadata with whole counting (a paper is credited to every country appearing in the affiliations of its authors); 7 of the 746 records had no country information and were not assigned to any country. Citations per year = citations/(2026 - publication year + 1). NCS = observed citations divided by the mean citations of all corpus records published in the same year; NCS > 1 means more cited than the same-year average. Both adjusted indicators are ratios computed within a single retrieval and are therefore insensitive to the retrieval date. 95% CIs are from 2,000 bootstrap resamples. SCP, single-country publications; MCP, multiple-country publications. Countries with fewer than five publications are not ranked.

In terms of total citations, the United States accumulated the most (7,112), followed by Switzerland (2,755), the United Kingdom (2,506), Italy (2,403) and China (2,031). After normalization for publication year, Switzerland (normalized citation score 1.72, 95% CI 1.15–2.37), Austria (1.69, 0.90–2.61), Italy (1.58, 1.10–2.16) and Canada (1.52, 0.99–2.10) had the highest citation impact, whereas China’s score (0.96, 0.74–1.21) was close to the corpus average; raw per-paper averages are not used for ranking because they are strongly influenced by publication age, document type and the number of publications.

### 3.3 Institutions

**Figure 4A** is a bar chart illustrating the publication output of institutions and presents the top 10 institutions ranked by publication volume. Amgen was the most productive institution (n = 47, 831 citations), followed by the University of Texas MD Anderson Cancer Center (n = 18) and the University of California, Los Angeles (n = 18), Clinique de Genolier (n = 17) and Hospital Clínic de Barcelona (n = 16) (**Table 2**). The institutional collaboration network (**Figure 4B**) had a multi-hub, core–periphery structure. Amgen, reported as a single corporate entity after merging four OpenAlex records, was the most productive institution (n = 47) and the largest and most connected node in the institutional collaboration network (15 collaborating institutions; total link strength = 33). Amgen was followed by The University of Texas MD Anderson Cancer Center (n = 18), the University of California, Los Angeles (n = 18), Clinique de Genolier (n = 17) and Hospital Clínic de Barcelona (n = 16) (**Table 2**). The network contained 35 institutions and 95 co-authorship links and resolved into six clusters. The largest cluster represented a US academic–industry group centered on Amgen, UCLA, the University of Pennsylvania, Penn Hospital, Georgetown University and United BioSource Corporation. Other clusters comprised major evidence-generating centers in North America and Europe; a European clinical–industry group; an Asia–Europe group including Sun Yat-sen University, the Catholic University of Korea, the University of Ulsan and the University of Barcelona; and smaller European university-hospital and UK academic–NHS collaborations. Amgen, UCLA, the University of Pennsylvania and Wilhelminenspital occupied the most central positions, whereas most other institutions had relatively few connections, indicating that institutional collaboration was concentrated around a limited number of hubs.

**Table 2:** Top 15 institutions by publication output on cancer-related anemia (2010-2025), with institution type and age-adjusted citation indicators.

Rank	Institution	Type	Country	Publications	Citations (OpenAlex)	Mean citations	Median citations	Citations per year	NCS	NCS CI low	NCS CI high
1	Amgen	company	CH;DE;GB;US	47	831	17.7	8	1.27	0.55	0.34	0.85
2	The University of Texas MD Anderson Cancer Center	healthcare	US	18	530	29.4	22.5	3.95	1.83	0.85	3.31
3	University of California, Los Angeles	education	US	18	1176	65.3	23.5	6.03	1.99	1.07	3.1
4	Clinique de Genolier	healthcare	CH	17	1182	69.5	39	6.37	2.01	1.1	3.1
5	Hospital Clínic de Barcelona	healthcare	ES	16	806	50.4	14	4.74	1.52	0.5	2.76
6	Sun Yat-sen University	education	CN	13	384	29.5	23	3.05	1.02	0.8	1.26
7	University of Pennsylvania	education	US	13	286	22	19	3.05	1.03	0.45	1.94
8	Wilhelminen Hospital	healthcare	AT	12	1251	104.2	39	8.27	2.98	1.25	4.91
9	University of Utah	education	US	11	840	76.4	45	8.08	2.85	1.44	4.39
10	Catholic University of Korea	education	KR	10	196	19.6	19	1.94	0.7	0.35	1.12
11	Istituti di Ricovero e Cura a Carattere Scientifico	healthcare	IT	10	404	40.4	12	5.17	1.62	0.31	3.47
12	Ghent University Hospital	healthcare	BE	9	82	9.1	8	0.76	0.31	0.21	0.42
13	Huntsman Cancer Institute	healthcare	US	9	722	80.2	20	8.32	3.09	1.4	4.99
14	Pennsylvania Hospital	healthcare	US	9	480	53.3	25	4.18	1.71	0.8	2.94
15	Sun Yat-sen University Cancer Center	healthcare	CN	9	342	38	31	3.51	1.12	0.86	1.41

Institutions are the OpenAlex institution entities of the authors’ affiliations; institutions belonging to the same corporate group were merged, so the four Amgen entities indexed by OpenAlex (United States, Switzerland, United Kingdom, Germany; 47 records in total) are reported as one company. Type is the OpenAlex institution type (company, education, healthcare, government, nonprofit). Citation values are from OpenAlex, retrieved 24 September 2026; the age-adjusted and normalized indicators follow the definitions in the footnote to **Table 1**. Institutions with fewer than five publications are not ranked.

**Figure 4:**
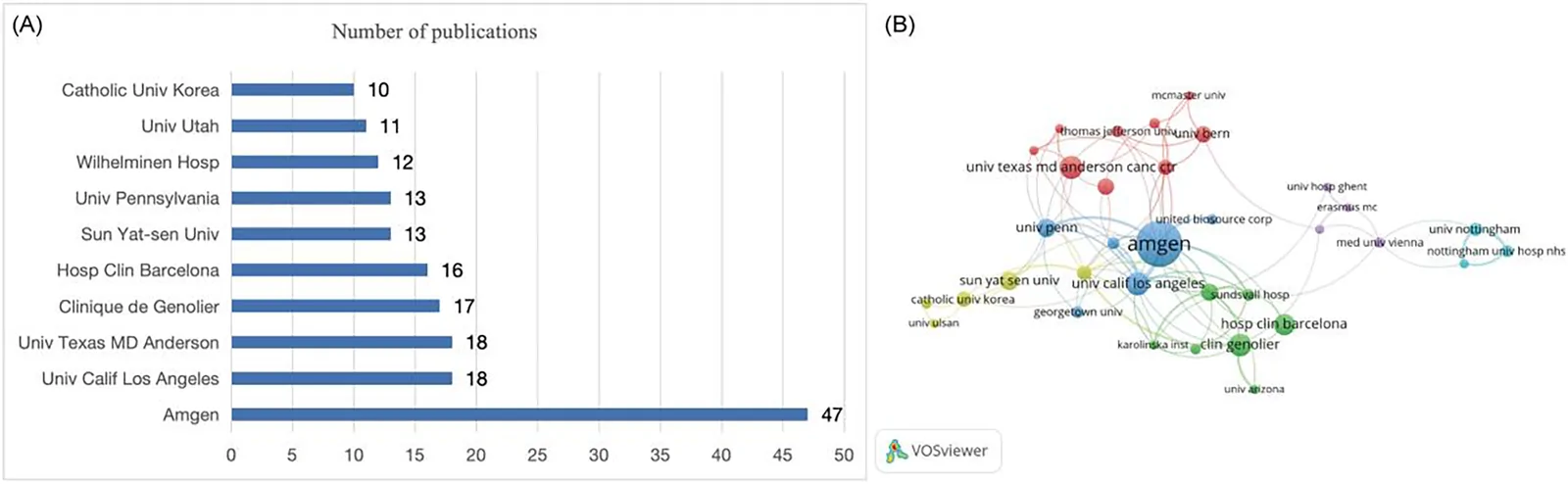
Productive institutions in cancer-related anemia research. **(A)** Top 10 institutions by publication count. **(B)** Institutional collaboration network constructed with VOSviewer (institutions appearing at least five times); node color represents detected communities.

### 3.4 Authors

**Figure 5A** is a bar chart of author publication counts, showing the top 10 authors by publication number. Aapro M was the most productive author (n = 9; h-index 9), followed by Bohlius J (n = 5; h-index 5), Rodgers G (n = 5; h-index 4) and Steinmetz T (n = 5; h-index 4) (**Table 3**). The author collaboration network (**Figure 5B**) showed a multi-core structure. A tightly connected European supportive-care cluster was built around Aapro, Gascón, Ludwig, Glaspy and Osterborg, the authors of the European Society for Medical Oncology (ESMO)/European Organisation for Research and Treatment of Cancer (EORTC) guidance and ESA trials; a US evidence- and guideline-oriented community centered on Bennett, Bohlius, Seidenfeld and Djulbegovic; and additional cores formed by US iron-therapy researchers (Auerbach, Henry, Rodgers and Steensma). Cross-cluster collaboration remained limited, indicating that CRA research is conducted largely within established teams.

**Table 3:** Top 15 authors by publication output on cancer-related anemia (2010–2025).

Rank	Author	Publications	Citations	Avg citations	h-index
1	Aapro M	9	787	87.44	9
2	Bohlius J	5	431	86.20	5
3	Rodgers G	5	312	62.40	4
4	Steinmetz T	5	151	30.20	4
5	Ludwig H	4	464	116.00	3
6	Keeler B	4	369	92.25	4
7	Glaspy J	4	302	75.50	4
8	Wilson M	4	230	57.50	4
9	Abdel-Razeq H	4	184	46.00	3
10	Laso-Morales M	4	117	29.25	4
11	Zhang L	4	82	20.50	3
12	Gascón P	4	43	10.75	3
13	Gilreath J	3	378	126.00	3
14	Henry D	3	127	42.33	3
15	Vadhan-Raj S	3	125	41.67	3

**Figure 5:**
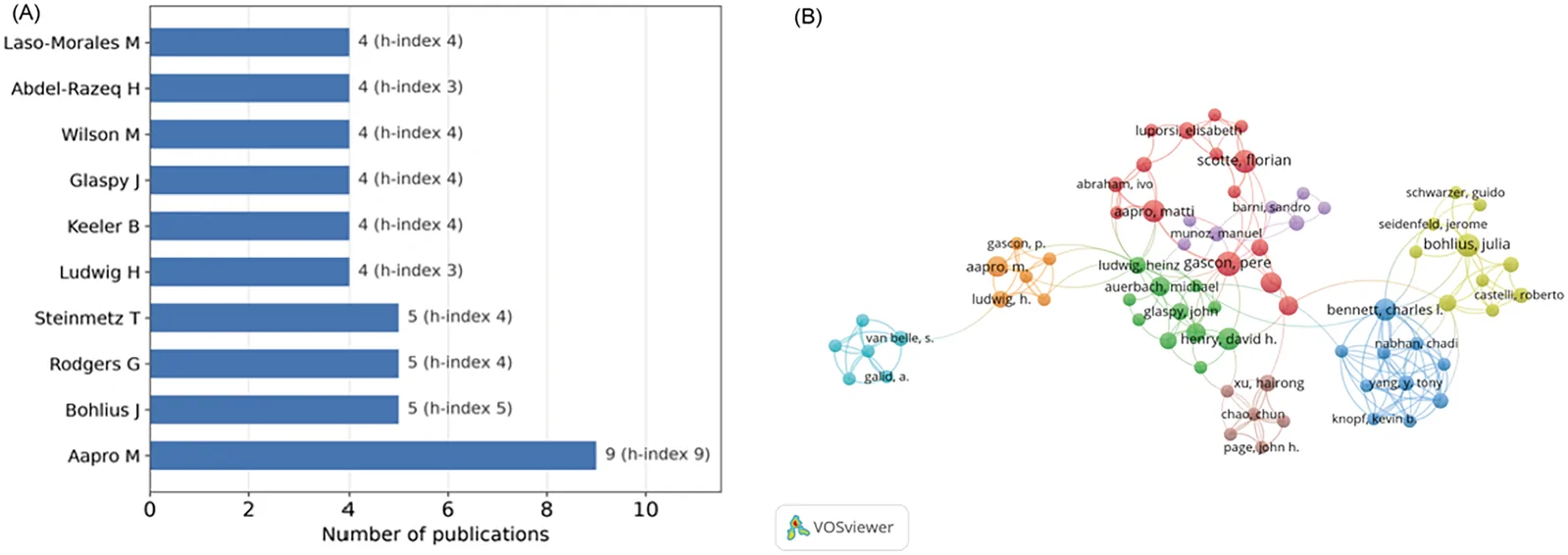
Productive authors in cancer-related anemia research. **(A)** Top 10 authors by publication count (with h-index). **(B)** Author collaboration network constructed with VOSviewer (authors appearing at least three times); node color represents detected communities.

### 3.5 Journals

**Figure 6A** is a bar chart showing the top 10 journals by publication count. Publications appeared in 392 journals. The top 10 journals by publication count (as ranked in **Table 4**) published 132 papers (17.7%). Supportive Care in Cancer was the most prolific journal (n = 44, 667 citations), followed by BMC Cancer (n = 14), Frontiers in Oncology (n = 12), Cureus (n = 11) and Annals of Oncology (n = 9). In terms of citation impact, Annals of Oncology had by far the highest normalized citation score (3.99, 95% CI 2.07–6.00; 1,255 citations from 9 papers), reflecting the influence of the ESMO clinical practice guidelines on CRA management, although this average is driven by a small number of highly cited guidelines and reviews and by the publication age of these articles; the Critical Reviews in Oncology/Hematology (NCS 2.09) and the American Journal of Hematology (mean 69.7 citations per paper) followed. The temporal pattern (**Figure 6B**) showed a broad, steady presence of supportive-care and oncology journals, with growth of Cureus and Frontiers in Oncology in recent years, consistent with increasing open-access output in the field.

**Table 4:** Journals with at least five publications in the corpus, ranked by publication output, with age-adjusted citation indicators anemia.

Rank	Journal	Publications	Citations (OpenAlex)	Mean citations	Median citations	Citations per year	NCS	NCS CI low	NCS CI high
1	SUPPORTIVE CARE IN CANCER	44	667	15.2	8.5	1.62	0.65	0.46	0.86
2	BMC CANCER	14	284	20.3	15	2.08	0.85	0.6	1.1
3	FRONTIERS IN ONCOLOGY	12	102	8.5	5.5	2.43	1.25	0.54	2.26
4	CUREUS	11	42	3.8	1	0.87	0.39	0.16	0.67
5	ANNALS OF ONCOLOGY	9	1255	139.4	151	10.68	3.99	2.07	6
6	JOURNAL OF THE NATIONAL COMPREHENSIVE CANCER NETWORK	9	354	39.3	19	3.18	1.31	0.43	2.64
7	PLOS ONE	9	207	23	22	2.77	1.02	0.72	1.31
8	ANTICANCER RESEARCH	8	39	4.9	4	0.59	0.24	0.1	0.42
9	CANCERS	8	70	8.8	5	1.86	0.92	0.64	1.28
10	CRITICAL REVIEWS IN ONCOLOGY HEMATOLOGY	8	283	35.4	17.5	5.38	2.09	0.58	4.09
11	CURRENT MEDICAL RESEARCH AND OPINION	8	48	6	7	0.4	0.22	0.14	0.29
12	ONCOLOGIST	8	297	37.1	29.5	2.41	1.36	0.68	2.36
13	AMERICAN JOURNAL OF HEMATOLOGY	7	488	69.7	28	5.68	2.28	0.99	4.22
14	CLINICAL & TRANSLATIONAL ONCOLOGY	7	91	13	14	1.3	0.63	0.28	1.1
15	CURRENT ONCOLOGY	7	102	14.6	6	2.09	0.7	0.3	1.12

Same methods as **Table 1**: journals are the names indexed in the source databases, normalized for case and punctuation differences between Web of Science and PubMed. Journals with fewer than five publications in the corpus are not ranked. NCS values above 1 indicate that the papers in a journal were cited more often than the average corpus paper published in the same year; the confidence intervals show that several apparent differences between journals are not resolved by the data.

**Figure 6:**
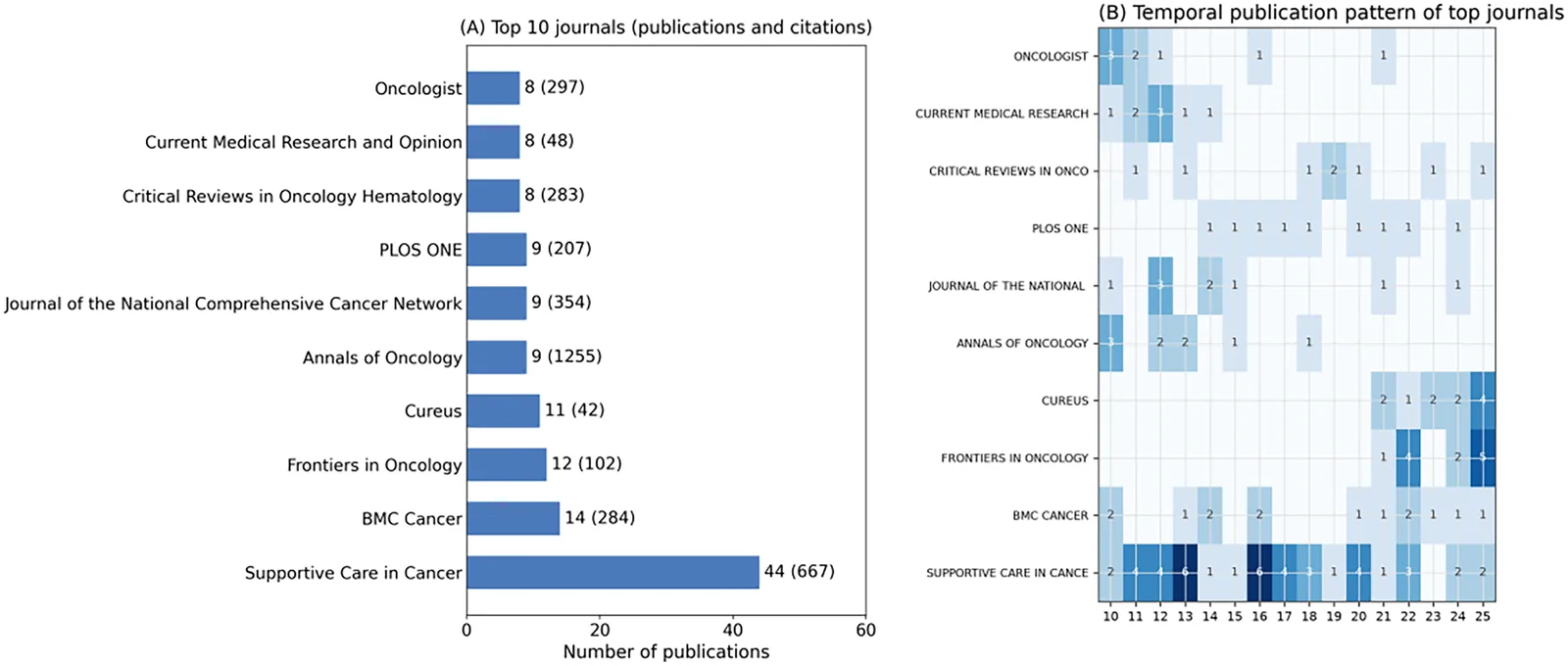
Core journals in cancer-related anemia research. **(A)** Top 10 journals by publication count (numbers in parentheses indicate citations; orange bars scaled to citations). **(B)** Temporal publication pattern of the top 10 journals, 2010–2025.

### 3.6 Citation analysis

The most-cited publications were predominantly systematic reviews and clinical guidelines (**Figure 7A**): the Cochrane systematic review of erythropoietin or darbepoetin for patients with cancer (391 citations) (22), the ASCO/ASH clinical practice guideline on epoetin and darbepoetin in cancer (305 citations) (23), the ESMO clinical practice guidelines for the management of anemia and iron deficiency in cancer (290 citations) (5), the study of iron deficiency prevalence across tumors (265 citations) (24), and the prospective observational study of inflammation, iron and nutrition in CRA (255 citations) (3). The diagnosis-and-treatment review of cancer-related anemia (248 citations) (1), the study-level meta-analysis of ESA safety outcomes (238 citations) (15) and the NCCN clinical practice guideline on cancer- and chemotherapy-induced anemia (228 citations) (25) were also highly cited. Among mechanism-focused works, the study of extramedullary erythropoiesis in late-stage tumors (200 citations) (26) and the analysis of inflammation-induced tryptophan breakdown in anemia, fatigue and depression (177 citations) (27) illustrate the field’s mechanistic grounding in iron metabolism and inflammation biology. The ASCO/ASH 2010 guideline appears twice in **Figure 7A** because the same content was dual-published in the Journal of Clinical Oncology (305 citations) and Blood (207 citations); both DOI records were retained and counted separately.

**Figure 7:**
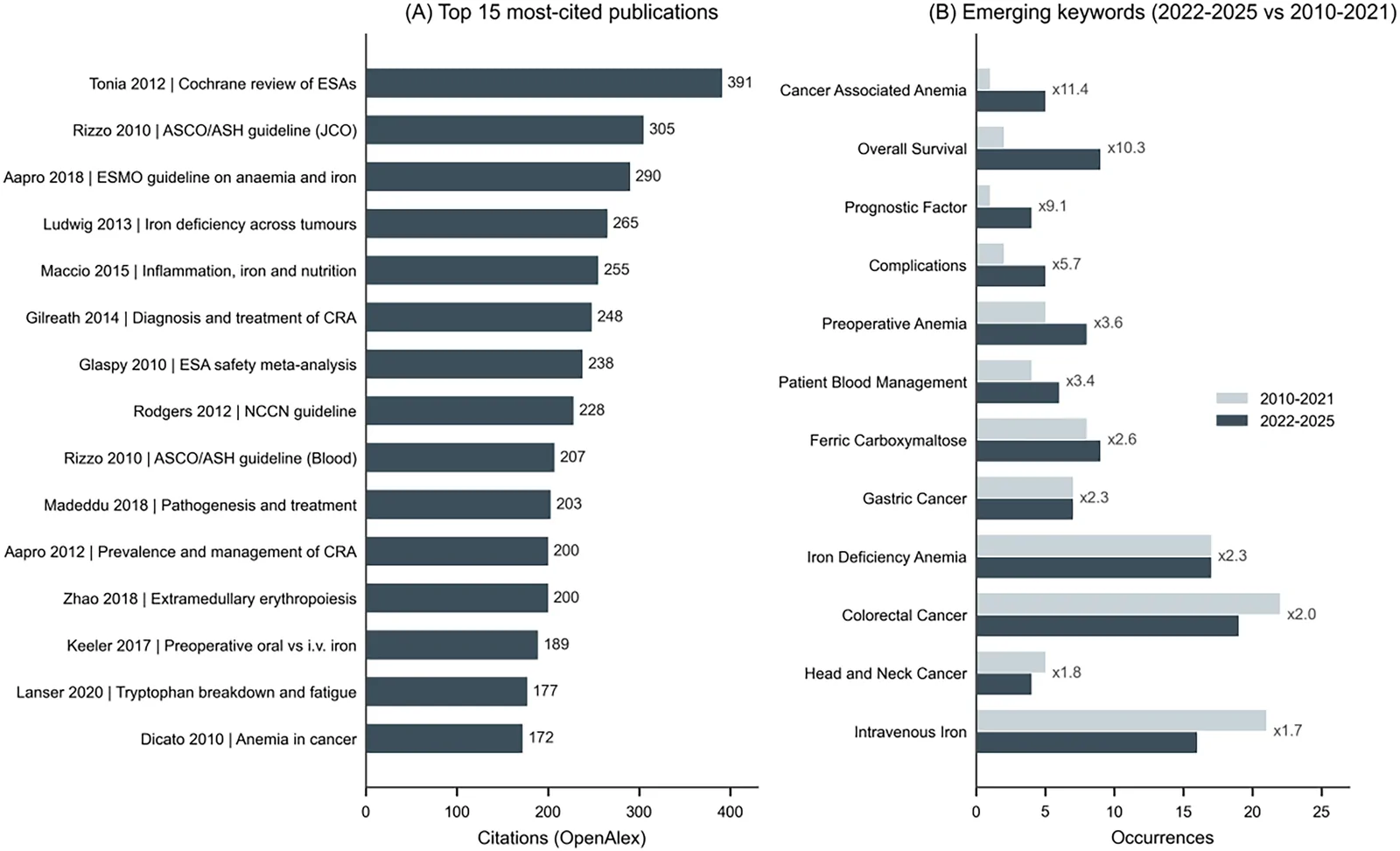
Citation impact and emerging research fronts. **(A)** Top 15 most-cited publications; labels give the first author and the year of publication, and bars show the number of citations recorded in OpenAlex (retrieved 24 September 2026). The 2010 ASCO/ASH guideline appears twice because the same content was dual-published in the Journal of Clinical Oncology and Blood; both DOI records are shown separately. **(B)** Author keywords whose share of all keyword occurrences in 2022–2025 was at least 1.6-fold higher than in 2010–2021 (with at least four occurrences after 2021); the value beside each pair is the ratio of the two period shares.

Reference co-citation analysis also delineated the intellectual base of the field (**Figure 8A**). The co-cited reference network was organized around two dominant, tightly connected clusters: an ESA evidence cluster built on landmark randomized trials and meta-analyses of epoetin and darbepoetin [Glaspy et al., 2010 (15); Bohlius et al., 2009 (28); Bennett et al., 2008 (29)] and a clinical-epidemiology and iron-deficiency cluster centered on prevalence surveys, iron-deficiency studies and updated management guidance [Ludwig et al., 2013 (24); Aapro et al., 2012 and 2018 (5, 13); Gilreath et al., 2020 (30)], together with a smaller treatment-practice cluster on ESA dosing and iron supplementation. Citation-burst analysis (**Figure 8B**) showed that the strongest recent bursts corresponded to the ESMO guidelines on anemia and iron deficiency in cancer [Aapro et al., 2018; strength 13.15, 2019–2023 (5)] and to contemporary CRA and iron-restriction reviews [Gilreath et al., 2020, Blood; strength 12.60, 2022–2025 (30); Abdel-Razeq and Hashem, 2020 (31)] The early bursts were uniformly ESA trials, confirming that the field’s intellectual base is shifting from ESA-centered evidence toward iron biology, perioperative blood management and updated clinical guidance.

**Figure 8:**
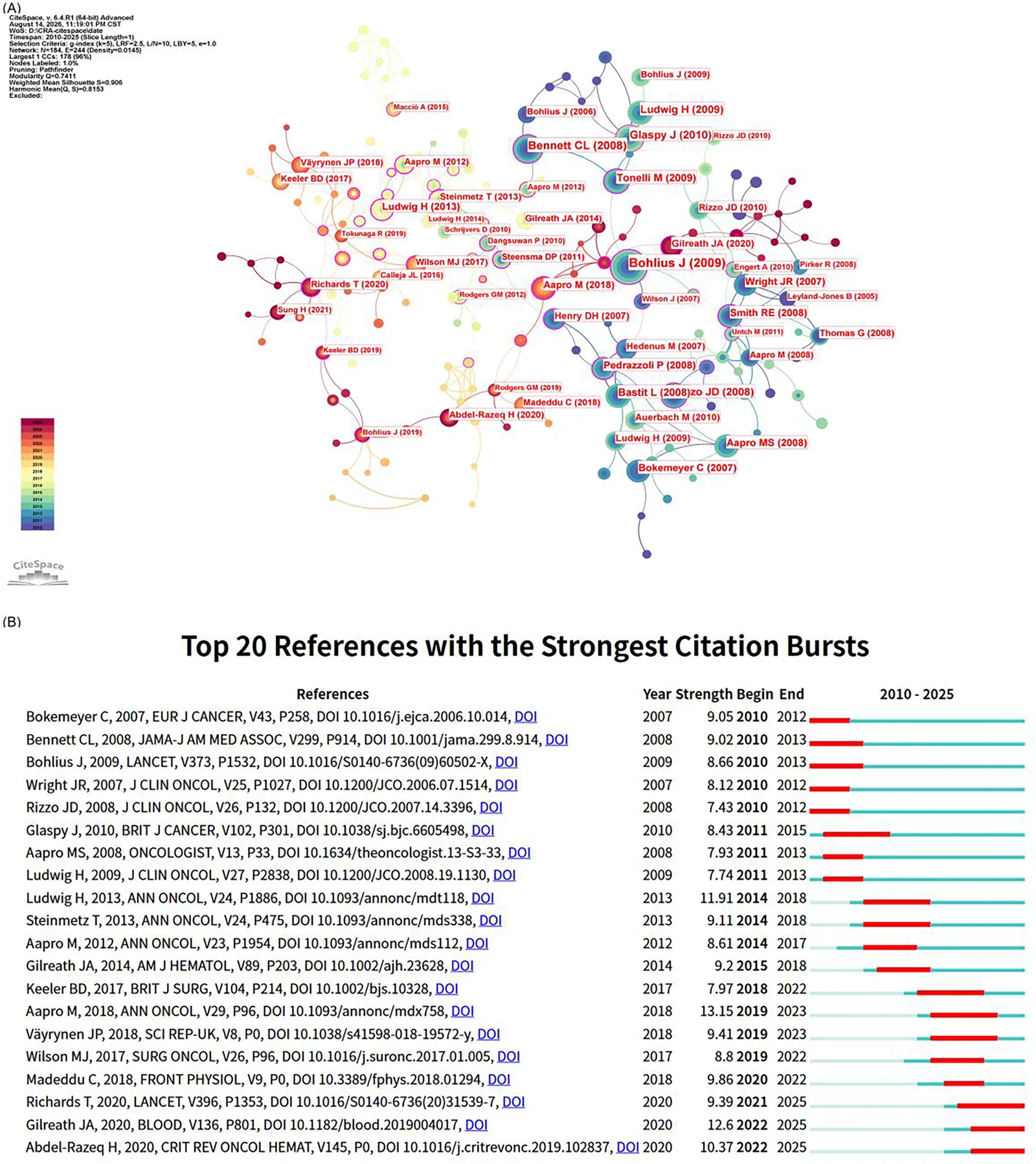
Intellectual base of cancer-related anemia research (CiteSpace). **(A)** Co-citation network of references; node size is proportional to co-citation frequency and colors denote clusters. **(B)** Top 20 references with the strongest citation bursts, 2010–2025; red segments indicate burst periods.

### 3.7 Keywords and research hotspots

After harmonization of spelling and synonym variants, the most frequent author keywords were anemia (n = 312), cancer (n = 76), erythropoiesis-stimulating agents (n = 74), chemotherapy-induced anemia (n = 65), hemoglobin (n = 59), chemotherapy (n = 55), erythropoietin (n = 48), colorectal cancer (n = 41), iron deficiency anemia (n = 39), intravenous iron (n = 37), prognosis (n = 36), survival (n = 35) and iron deficiency (n = 34) (**Table 5**). These frequencies are based on the harmonized keyword dictionary used for the VOSviewer network; therefore, anemia, hemoglobin and related spelling or synonym variants are not counted separately.

**Table 5:** Top 25 normalized author keywords in cancer-related anemia research (2010–2025).

Rank	Author keyword	Frequency
1	Anemia	312
2	Cancer	76
3	Erythropoiesis-Stimulating Agents	74
4	Chemotherapy-Induced Anemia	65
5	Hemoglobin	59
6	Chemotherapy	55
7	Erythropoietin	48
8	Colorectal Cancer	41
9	Iron Deficiency Anemia	39
10	Intravenous Iron	37
11	Prognosis	36
12	Survival	35
13	Iron Deficiency	34
14	Cancer-Related Anemia	29
15	Darbepoetin Alfa	27
16	Transfusion	27
17	Hepcidin	26
18	Breast Cancer	24
19	Iron	23
20	Quality Of Life	23
21	Blood Transfusion	22
22	Biosimilars	17
23	Ferric Carboxymaltose	17
24	Fatigue	14
25	Gastric Cancer	14

Frequencies are based on the harmonized keyword dictionary used for the VOSviewer co-occurrence analysis. Spelling and synonym variants were merged before counting.

The normalized keyword network (**Figure 9A**) resolved into several color-coded communities that could be grouped into three broad themes. The treatment and management theme was characterized by erythropoiesis-stimulating agents, darbepoetin alfa, chemotherapy-induced anemia, iron therapy, iron supplementation and patient blood management. The anemia and iron-related mechanism theme included iron deficiency, iron deficiency anemia, hemoglobin, ferritin, inflammation, hepcidin, erythropoiesis and erythropoietin receptor. The cancer and clinical-outcome theme comprised colorectal, breast, lung, gastrointestinal, ovarian and head and neck cancers, together with surgery, preoperative anemia, transfusion, prognosis, survival, mortality and quality of life. The density map (**Figure 9B**) showed the strongest co-occurrence density around anemia, cancer, erythropoiesis-stimulating agents, chemotherapy-induced anemia, darbepoetin alfa and iron-related terms. In the overlay map (**Figure 9C**), earlier terms (blue, approximately 2016) included erythropoiesis-stimulating agents, darbepoetin alfa, chemotherapy-induced anemia and cancer, whereas later terms (yellow/red, approximately 2020) included iron deficiency anemia, iron therapy, iron supplementation, patient blood management, preoperative anemia, colorectal cancer, prognosis and quality of life.

**Figure 9:**
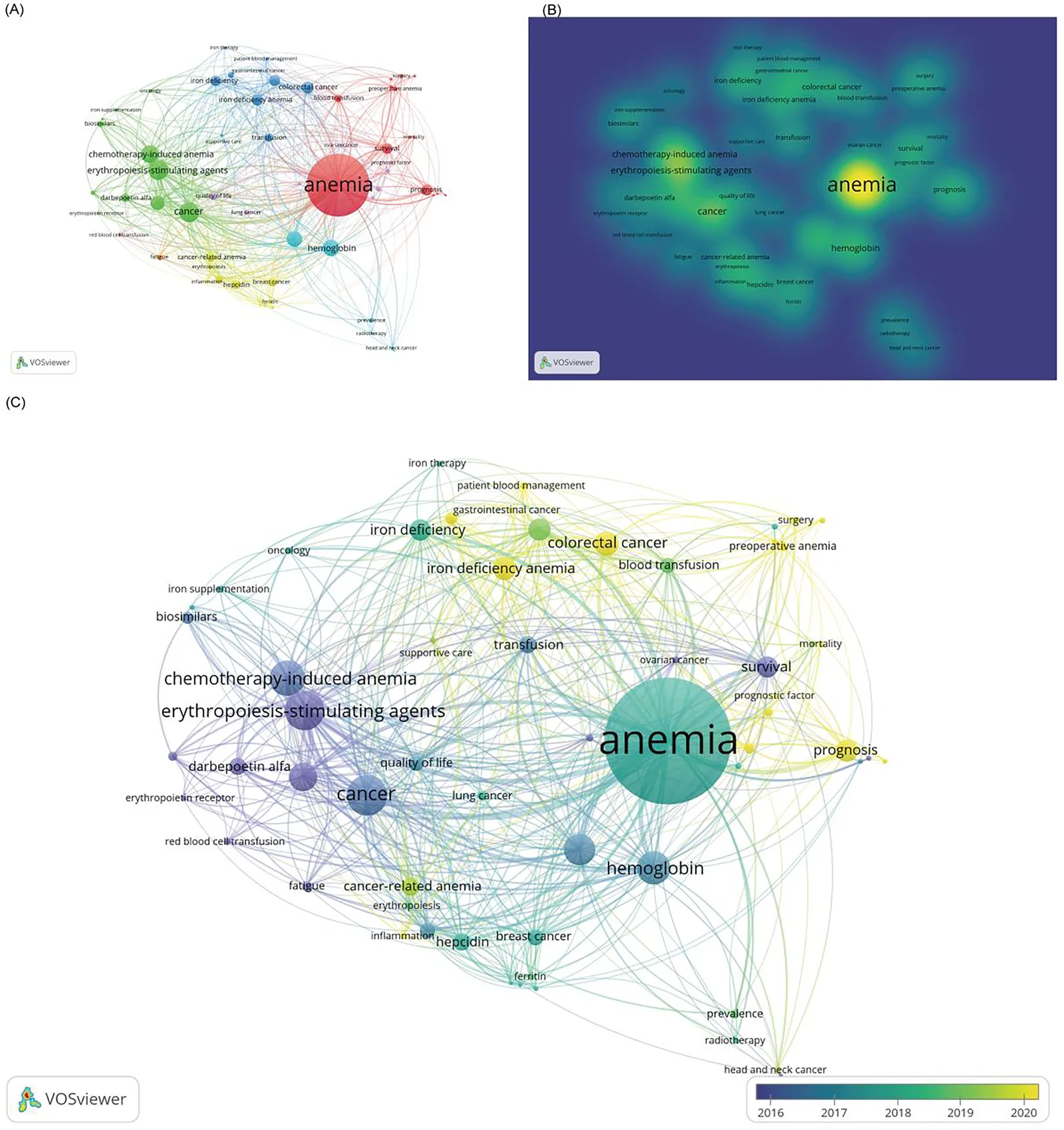
Keyword structure and hotspots. **(A)** Keyword co-occurrence network constructed with VOSviewer (keywords appearing at least five times); node size is proportional to occurrence frequency and colors denote communities. **(B)** Keyword density map constructed with VOSviewer; red-to-yellow regions indicate the highest co-occurrence density and represent the field’s research hotspots. **(C)** Keyword overlay map constructed with VOSviewer; node color indicates the average publication year from blue (earlier) to yellow (later).

The CiteSpace keyword timeline (**Figure 10A**) comprised 12 clusters, with representative themes including treatment outcome, C-reactive protein, intravenous iron, erythropoiesis-stimulating agents, iron deficiency, darbepoetin alfa, colorectal cancer, chemotherapy-induced anemia, chemotherapy, cancer-related anemia and anemia. Early research was concentrated in ESA- and clinical-trial-related terms. The subsequent period showed increasing attention to iron deficiency, preoperative anemia, colorectal cancer, transfusion and outcome-related terms. These patterns describe changes in keyword prominence and research attention rather than changes in clinical practice. These patterns describe a change in the relative prominence of keywords rather than a change in clinical practice: research attention moved from single-agent ESA-related topics toward iron-related, perioperative and outcome-oriented terms. In the period-normalized comparison, the highest 2022-2025/2010-2021 keyword share ratios were observed for cancer-associated anemia (11.4), overall survival (10.3), prognostic factor (9.1), complications (5.7), preoperative anemia (3.6) and patient blood management (3.4) (**Figure 7B**). The choice of threshold does not change the main fronts. At cut-offs of 1.5, 1.6, 2.0, 2.5 and 3.0 the number of keywords selected was 14, 12, 9, 7 and 6, respectively (**Supplementary Table S2**). Cancer-associated anemia, overall survival, prognostic factor, complications, preoperative anemia and patient blood management were retained at every threshold; ferric carboxymaltose was retained up to a cut-off of 2.5, and iron deficiency anemia and gastric cancer up to 2.0; colorectal cancer, head and neck cancer, intravenous iron and breast cancer qualified only at the two lowest cut-offs, and surgery only at 1.5.

**Figure 10:**
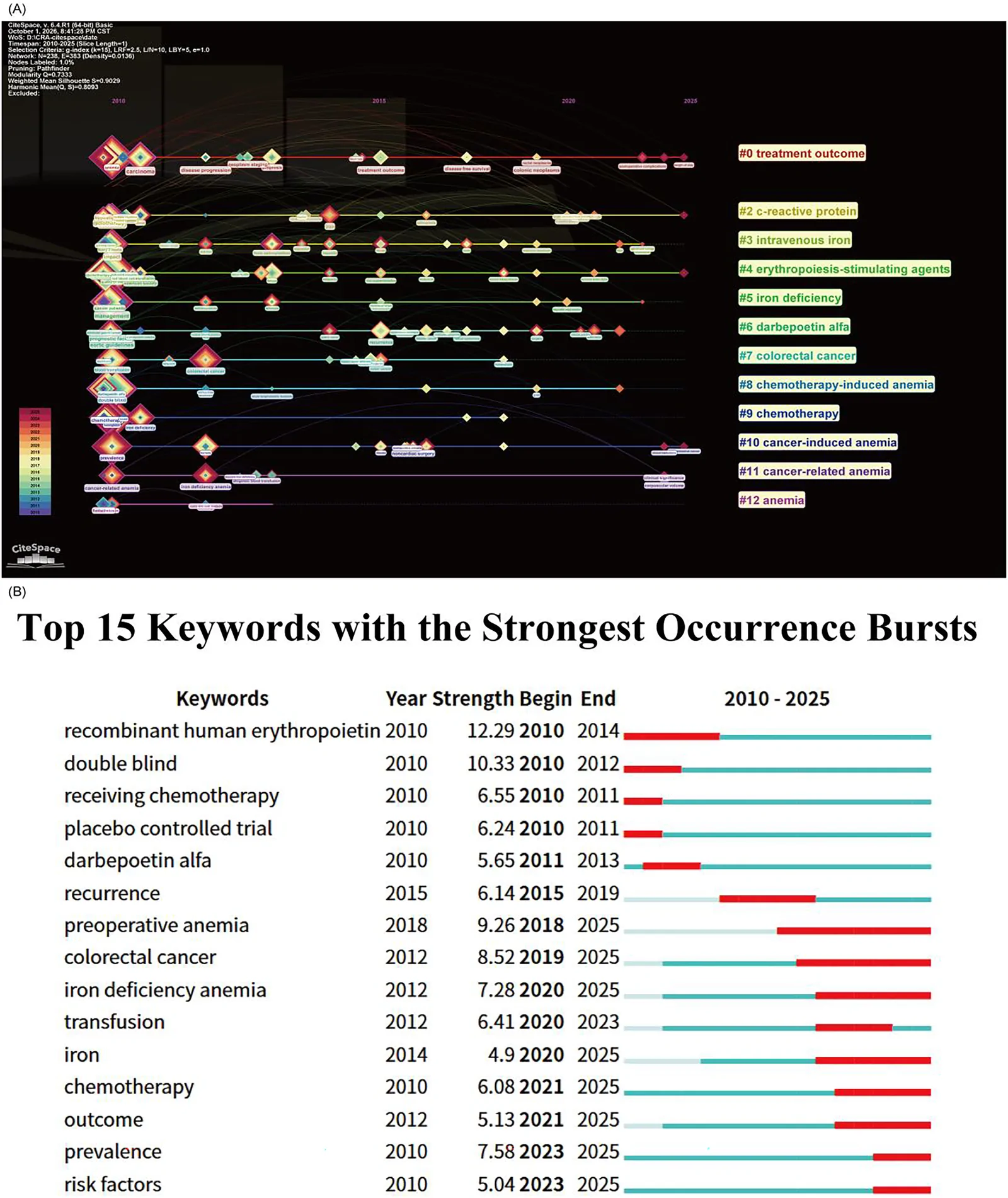
Temporal evolution of keyword research hotspots (CiteSpace 6.4.R1). **(A)** Keyword timeline based on one-year time slices, comprising 238 nodes, 383 links and 12 clusters; nodes are positioned by year of first appearance and colored by cluster. **(B)** Top 15 keywords with the strongest occurrence bursts, 2010–2025; red segments indicate periods of increased keyword occurrence.

Keyword occurrence-burst analysis (**Figure 10B**) identified three temporal patterns. The initial burst wave comprised recombinant human erythropoietin (strength 12.29, 2010–2014), double blind (10.33, 2010–2012), receiving chemotherapy (6.55, 2010–2011), placebo controlled trial (6.24, 2010–2011) and darbepoetin alfa (5.65, 2011–2013). A mid-period burst centered on recurrence (6.14, 2015–2019). The later and ongoing wave comprised preoperative anemia (9.26, 2018–2025), colorectal cancer (8.52, 2019–2025), iron deficiency anemia (7.28, 2020–2025), transfusion (6.41, 2020–2023), iron (4.90, 2020–2025), chemotherapy (6.08, 2021–2025), outcome (5.13, 2021–2025), prevalence (7.58, 2023–2025) and risk factors (5.04, 2023–2025). These bursts indicate a transition from ESA- and trial-centered terminology toward iron deficiency, perioperative care, colorectal cancer and outcome-oriented research.

## 4 Discussion

### 4.1 Principal findings

This study presents a comprehensive bibliometric analysis of cancer-related anemia that combines Web of Science and PubMed records across the 2010–2025 period, complementing existing narrative and mechanism-specific reviews; the scope and interpretation of the analysis are bounded by the limitations discussed in Section 4.5. We identified 746 unique English-language articles and reviews and showed that annual output was stationary at about 45 publications per year between 2010 and 2024 (annual rate ratio 1.00, 95% CI 0.98–1.02), with a single-year increase to 70 publications in 2025. The field is characterized by: (i) a geographically distributed structure in which the United States leads in output and citations, China and other Asian countries contribute large volumes, and several small European countries achieve the highest year-normalized citation scores; (ii) a strong industry–academic axis, with Amgen as the most productive institution and ESA-related topics dominating the early literature; (iii) in keyword frequencies, a movement of research attention from ESA-centered topics toward mechanism-based iron biology, perioperative patient blood management and outcome-oriented research.

### 4.2 Geographic and collaboration patterns

The United States’ leadership is consistent with its historical role in developing ESA and iron therapies and in generating clinical practice guidelines (23). China’s second-place output reflects its large cancer burden and expanding supportive-care research capacity. Its year-normalized citation score was close to the corpus average (0.96, 95% CI 0.74–1.21), so output volume and citation impact should not be compared directly on these data. The high year-normalized citation scores of Switzerland, Austria and Italy (1.72, 1.69 and 1.58) may reflect the concentration of European leaders in CRA research, including the EORTC/ESMO supportive-care networks, together with the publication of landmark guidelines and mechanism-based studies in these countries (5, 24); the confidence intervals overlap, so these differences should be read as indicative. The collaboration network shows dense transatlantic ties and visible US–China collaboration links (**Figure 3B**), although the temporal evolution of this axis was not quantified in the present analysis. The share of internationally co-authored publications is markedly lower among leading Asian countries (China, 20/105 [19.0%]; Japan, 10/41 [24.4%]; South Korea, 6/40 [15.0%]) than among leading European countries (Switzerland, 45/50 [90.0%]; Germany, 43/64 [67.2%]; United Kingdom, 42/77 [54.5%]; **Table 1**), indicating that cross-border collaboration within Asia remains an opportunity for future research consortia.

### 4.3 Hotspots and thematic evolution

The three broad thematic domains identified from the normalized keyword network—treatment and clinical management, iron-related mechanisms, and cancer-specific clinical outcomes—correspond to the clinical architecture of CRA care. The management cluster reflects the long-standing centrality of ESAs and, increasingly, of intravenous iron: functional iron deficiency is now recognized as a major cause of ESA hyporesponsiveness, and trials of ferric carboxymaltose and iron isomaltoside have established iron repletion as a first-line component of CRA management (5, 13), in line with cross-indication guidance on iron deficiency (32) and with systematic evidence on iron therapy in anemic adults (33). The mechanistic cluster points to hepcidin as the central regulator of the anemia of inflammation: tumor-derived and treatment-related cytokines such as IL-6 and TNF-α stimulate hepcidin, sequester iron in macrophages and limit erythropoiesis (3, 34–36). The clinical-epidemiology cluster points to growing attention to tumor-specific anemia burden, particularly colorectal, gastric, breast and lung cancers, and to the prognostic value of anemia, consistent with evidence that preoperative anemia and transfusion independently predict postoperative complications and survival (37, 38).

The corpus is dominated by treatment-oriented terms, whereas bibliometric analyses that focus on symptom burden and patient-reported outcomes address a literature that our search does not capture. Two factors plausibly contribute to the change in keyword prominence: the availability of high-dose intravenous iron, which allows rapid repletion in chemotherapy and surgical settings (33, 39), and the wider adoption of patient blood management programs in surgical oncology (16). These observations describe a change in research attention; they cannot show whether clinical outcomes improved over the same period.

### 4.4 Emerging frontiers

Consistent with the keyword-burst and emerging-front analyses (**Figures 7B**, **10B**), four fronts are directly visible in the corpus. First, perioperative anemia and patient blood management, driven by evidence that systematic screening, iron repletion and transfusion stewardship improve surgical outcomes in gastrointestinal and gynecologic oncology (16, 39). Guidelines recommend hemoglobin assessment and iron repletion before major surgery, and the results of the PREVENTT trial (39) have kept open the question of which patients benefit from preoperative iron. Second, the growing use of high-dose intravenous iron, including ferric carboxymaltose, which reflects a move toward rapid and complete iron repletion whose efficacy and safety are still under evaluation in real-world cohorts (33). Single-visit repletion also simplifies treatment in chemotherapy and surgical pathways. Third, tumor-type-specific outcome research, particularly in colorectal, gastric and head neck cancers. Anemia at diagnosis and during treatment is associated with poorer outcomes in these tumor types (37, 38), and studies of them increased after 2018. Fourth, outcome- and risk-oriented terms, particularly recurrence, outcome, prevalence and risk factors, which entered their strongest burst periods after 2015. Survival and complication endpoints have become standard endpoints in oncology trials and now appear more often in the CRA literature.

Three clinically salient topics were not identified by the keyword analysis and cannot be evaluated from the corpus; they are raised here as directions reported in the recent literature. Microangiopathic hemolytic anemia is a rare paraneoplastic presentation that is documented in case series but was retrieved in insufficient numbers by our query (40). Roxadustat and other hypoxia-inducible factor prolyl hydroxylase inhibitors are indexed under different terminology and are represented by very few records, although they have been evaluated for chemotherapy-induced anemia in a phase 2 study and a randomized active-control trial (41, 42). Finally, immune-mediated cytopenias, including immune hemolytic anemia associated with immune checkpoint inhibitors, are documented in the literature (12, 17, 43) but were excluded from this search by design, because they are distinct hematologic entities (Section 2.2); their clinical relevance is therefore not measurable in this corpus and should be assessed in dedicated reviews.

The keyword analysis measures how often terms are used, not how strong the underlying evidence is. A term may become frequent because a question is being studied intensively, and a settled question may produce few new publications. The fronts above should therefore be read as directions in which research attention is currently growing, and each requires assessment against the primary literature.

### 4.5 Strengths and limitations

This study has several strengths: an explicit and reproducible search strategy; rigorous screening that excluded distinct hematologic entities, off-topic records and non-article document types; integration of Web of Science and PubMed metadata with OpenAlex citation data; and a combination of descriptive, network and temporal analyses. Several limitations should be acknowledged. First, citation counts from OpenAlex may differ from Web of Science counts, and very recent publications have had limited time to accrue citations. Second, country and institution assignment relied on OpenAlex metadata, which occasionally aggregates sub-institutions. Third, keyword-based clustering is inherently subject to normalization choices, and MeSH indexing lags for very recent papers. Finally, the 2025 value is a single annual observation and may be influenced by changes in journal output and open-access publishing; incomplete indexing of late-2025 records in Web of Science would, if anything, lead to underestimation of the 2025 count, and differences in indexing speed between the two databases may affect recent-year counts. The analysis should be updated as additional records are indexed. The search was restricted to English-language records indexed in the Web of Science Core Collection and PubMed, which may under-represent research published in other languages or indexed only in other databases; the trends and networks reported here therefore describe this corpus. The contribution of this study is the harmonized dual-database corpus and the age-adjusted indicators rather than novelty of the topic, since related bibliometric analyses of adjacent topics, such as anemia and quality of life in patients with cancer, have been published. Citation counts are a snapshot retrieved on 24 September 2026 and will increase with time; the age-adjusted and year-normalized indicators reported in **Tables 1**, **2** and **4** are relative to the present corpus rather than to an external field baseline, and one record without a DOI or PubMed identifier could not be matched in OpenAlex and is excluded from the citation-based analyses.

### 4.6 Implications

The results point to three areas where evidence remains thin: collaboration between Asian countries, the translation of iron-regulatory mechanisms into trials, and supportive-care research in under-represented regions. Guidelines and trials are also concentrated at a small number of institutions, which suggests a role for international multicenter networks (5, 23). These are implications for research activity, not clinical recommendations: publication and keyword frequencies record what is being studied, not what is effective, and clinical practice for cancer-related anemia should continue to follow current guidelines.

## 5 Conclusion

Cancer-related anemia research output was stable at about 45 publications per year between 2010 and 2024 and reached a single-year maximum of 70 publications in 2025, and the field is becoming more mechanism-based, multimodal and patient-centered. The United States, China and the United Kingdom lead publication output, while several European countries achieve the highest citation impact. As reflected in keyword frequencies, research attention has moved from erythropoiesis-stimulating agent-centered topics toward iron metabolism and hepcidin biology, perioperative anemia and patient blood management, and tumor-specific prognostic research. Future work should strengthen international collaboration, translate iron-regulatory mechanisms into clinical trials, and evaluate therapeutic options that fall outside the keyword scope of this analysis in dedicated studies.

## Data Availability

The original contributions presented in the study are included in the article/**Supplementary Material**. Further inquiries can be directed to the corresponding author.
