# Supplementary material for: Global research trends and hotspots in cancer-related anemia from 2010 to 2025: a multidatabase bibliometric analysis
Source: Front Oncol. 2026 Oct 28;16:1971034. doi: 10.3389/fonc.2026.1971034 (PMC13751477; doi:10.3389/fonc.2026.1971034)
Supplement: Supplementary file 1 [file Table1.docx]

**Supplementary Table S1. Number of records at each stage of the literature search, screening and deduplication, separately for the WoSCC and PubMed**

| **Stage** | **Step** | **WoSCC** | **PubMed** | **Total** | **Exclusion reasons** |
| --- | --- | --- | --- | --- | --- |
| Identification | Records returned by the database query | 1,110 | 1,458 | 2,568 |  |
| Stage 1a | Removed by field limits | 10 | 19 | 29 | WoSCC: 10 records published outside 2010-2025. PubMed: 19 records that were non-article type, non-English or outside the window. |
| Stage 1a | Records screened on title and abstract | 1,100 | 1,439 | 2,539 |  |
| Stage 1b | Excluded at title/abstract screening | 535 | 545 | 1,080 | Distinct haematological entities (E4): 287 WoSCC, 404 PubMed, 691 total. Anaemia incidental, off topic or non-article type (E5): 248 WoSCC, 141 PubMed, 389 total. |
| Stage 1b | Retained after database-specific screening | 565 | 894 | 1,459 |  |
| Stage 2 | Duplicates removed |  |  | 509 | Identified by identifier matching: 509 (PMID 505, DOI 4). Identified by title-based matching: 0. |
| Stage 2 | Retained after deduplication |  |  | 950 |  |
| Stage 3 | Removed at record-level verification |  |  | 204 | Case reports 47 (E8); letters or other non-article types 2 (E9); records excluded in the corresponding copy of the other database 155 (E10; 38 titles contained a haematological entity term and 117 matched the exclusion list of the other database). 47 + 2 + 155 = 204, so no separate retraction category was needed at this stage. |
| Final | Included in the bibliometric analysis | 564 | 182 | 746 | Indexed in both databases 509; WoSCC only 55; PubMed only 182. Articles 632 (including 2 conference proceedings papers) + reviews 114. |

Step 1a removed records that did not meet the document-type, language or publication-year limits (WoSCC, n = 10 records published outside 2010-2025; PubMed, n = 19 records that were non-article document types, not in English or outside the window). Step 1b applied the inclusion and exclusion criteria in Section 2.2 to titles and abstracts, separately for the two databases. Duplicates were identified with a four-step algorithm (normalised DOI, PubMed identifier, WoSCC accession number and, for records without these identifiers or still unmatched, a normalised title + first author + year key with token-based title comparison). At Stage 3 every remaining record was re-examined against the complete record because document-type labels are not applied consistently by either database. Exclusion reason codes are defined in Supplementary File S1.
