# Supplementary material for: Global research trends and hotspots in cancer-related anemia from 2010 to 2025: a multidatabase bibliometric analysis
Source: Front Oncol. 2026 Oct 28;16:1971034. doi: 10.3389/fonc.2026.1971034 (PMC13751477; doi:10.3389/fonc.2026.1971034)
Supplement: Supplementary file 2 [file Table2.docx]

**Supplementary Table S2. Sensitivity of the emerging-front keyword selection to the fold-change cut-off (2010–2025)**

| **Threshold** | **Keywords selected (n)** | **Keywords (ratio)** |
| --- | --- | --- |
| 1.5 | 14 | Surgery (1.52); Breast cancer (1.63); Intravenous iron (1.74); Head and neck cancer (1.82); Colorectal cancer (1.97); Iron deficiency anemia (2.28); Gastric cancer (2.28); Ferric carboxymaltose (2.56); Patient blood management (3.42); Preoperative anemia (3.65); Complications (5.70); Prognostic factor (9.11); Overall survival (10.25); Cancer-associated anemia (11.39) |
| 1.6 (primary) | 12 | Intravenous iron (1.74); Head and neck cancer (1.82); Colorectal cancer (1.97); Iron deficiency anemia (2.28); Gastric cancer (2.28); Ferric carboxymaltose (2.56); Patient blood management (3.42); Preoperative anemia (3.65); Complications (5.70); Prognostic factor (9.11); Overall survival (10.25); Cancer-associated anemia (11.39) |
| 2.0 | 9 | Iron deficiency anemia (2.28); Gastric cancer (2.28); Ferric carboxymaltose (2.56); Patient blood management (3.42); Preoperative anemia (3.65); Complications (5.70); Prognostic factor (9.11); Overall survival (10.25); Cancer-associated anemia (11.39) |
| 2.5 | 7 | Ferric carboxymaltose (2.56); Patient blood management (3.42); Preoperative anemia (3.65); Complications (5.70); Prognostic factor (9.11); Overall survival (10.25); Cancer-associated anemia (11.39) |
| 3.0 | 6 | Patient blood management (3.42); Preoperative anemia (3.65); Complications (5.70); Prognostic factor (9.11); Overall survival (10.25); Cancer-associated anemia (11.39) |

*Author keywords were extracted from the 746 included records (574 records carried author keywords; the remainder contribute to the publication counts only). The value in parentheses is the ratio of the keyword's share of all keyword occurrences in 2022–2025 (n = 877) to its share in 2010–2021 (n = 1,998). Only keywords with at least four occurrences after 2021 were eligible. The cut-off used in the main analysis was 1.6.*
